# When tics are not the whole story: rethinking clinical assessment in children with tic disorders

**DOI:** 10.3389/fneur.2026.1971225

**Published:** 2026-10-21

**Authors:** Francesca Magostini, Alessandro Capuano

**Affiliations:** Child Neurology and Psychiatry, Azienda Sanitaria Locale Viterbo, Viterbo, Italy

**Keywords:** clinical assessment, functional impairment, neurodevelopment, neurological soft signs, premonitory urges, sensory processing, tic disorders, Tourette syndrome

## Abstract

Tic disorders are heterogeneous neurodevelopmental conditions in which observable tic severity does not necessarily reflect the difficulties that most affect the child’s everyday life. Premonitory sensations, pain, suppression effort, psychiatric symptoms, motor or sensory features, and executive vulnerabilities may contribute differently to clinical presentation and functional outcomes. In this Perspective, we propose a three-domain clinical framework that distinguishes: (1) observable tic phenomenology and severity; (2) subjective tic-related experience; and (3) the broader neurodevelopmental and psychiatric profile. The framework is intended as a clinical heuristic for organizing assessment and prioritization rather than as a validated dimensional model. Functional impairment, participation, adaptive functioning, and health-related quality of life are considered transversal outcomes through which the clinical relevance of each domain should be evaluated. We argue that assessment should remain selective and focused on findings with meaningful functional consequences. Clinical priorities should therefore be determined not by tic visibility alone, but by the modifiable factors most strongly associated with meaningful functional consequences. This approach may support more individualized, developmentally informed, and child-centered treatment goals while providing a framework whose clinical usefulness can be evaluated empirically.

## 1 Introduction

Tic disorders, including Tourette syndrome, persistent (chronic) motor or vocal tic disorder, and provisional tic disorder, are neurodevelopmental conditions characterized by sudden, rapid, recurrent, non-rhythmic movements or vocalizations with onset during childhood or adolescence (1, 2). Tics may be motor or phonic, simple or complex, and their expression can vary considerably in frequency, intensity, distribution, and complexity over time and across contexts (2). This intrinsic variability makes any single clinical observation necessarily incomplete.

Clinical assessment has traditionally and appropriately focused on tic phenomenology and observable severity. Characterizing tic repertoire and quantifying number, frequency, intensity, complexity, and interference remain essential for diagnosis, differential diagnosis, monitoring, and treatment decisions (2). However, the most visible manifestation of a tic disorder is not necessarily its most clinically informative one. Tic severity does not invariably coincide with overall illness severity, functional impairment, or the burden experienced by the child. The distinction between tic severity and broader illness severity was recognized early (3), and contemporary clinical guidelines already recommend assessment extending beyond tic phenomenology to include psychiatric comorbidity, cognitive, emotional, motor, psychosocial functioning, and quality of life (2). More recent work likewise indicates that quality of life and everyday functioning are shaped by psychiatric, psychosocial, and neurodevelopmental features in addition to tic severity itself (4, 5). Thus, a child with frequent and conspicuous tics may remain well adapted, whereas another with relatively subtle motor manifestations may experience considerable difficulties that are barely apparent during a clinical consultation.

This discrepancy becomes easier to understand when tics are considered not as isolated motor events but as part of a broader neurodevelopmental phenotype. Contemporary models implicate distributed cortico-striato-thalamo-cortical networks and their interactions with motor, prefrontal, limbic, and cerebellar systems involved in action selection, inhibitory control, sensorimotor integration, and learning (6, 7). This distributed architecture is consistent with the marked clinical heterogeneity of tic disorders and suggests that severity represents only one component of a broader clinical phenotype.

The internal experience of tics further illustrates this mismatch between what is observed and what is clinically relevant. Premonitory sensations, feelings of incompleteness or “not just right” experiences, pain, and the effort associated with tic suppression may contribute substantially to the child’s subjective experience. Tic suppression itself is neither an all-or-none ability nor a simple marker of voluntary control; it varies across individuals, tics, contexts, and time, and observable suppression may conceal rather than eliminate the effort associated with the disorder (2, 7, 8). In younger children, limited awareness or verbalization of premonitory sensations may further widen the gap between external observation and internal experience.

Tic disorders also occur within a broader neurological, neurodevelopmental, and psychiatric context. Psychiatric comorbidities are well recognized, while motor coordination, neurological soft signs, sensory processing, and executive functioning may represent additional clinically relevant characteristics that do not necessarily parallel tic severity. Their clinical significance, however, should be distinguished from their functional consequences: the presence of a symptom, trait, examination finding, or performance deficit does not by itself establish impairment in everyday life.

Against this background, we propose a three-domain clinical framework intended as a clinical heuristic rather than a new dimensional model of tic disorders. The proposed domains comprise: (1) observable tic phenomenology and severity; (2) subjective tic-related experience; and (3) the broader neurodevelopmental and psychiatric profile. Functional impairment, participation, adaptive functioning, and quality of life are considered transversal outcomes through which the clinical relevance of each domain can be evaluated. The novelty of this approach lies not in multidimensional assessment itself, which is already embedded in contemporary clinical guidance, but in explicitly separating these levels and relating them to real-life functioning for clinical prioritization. We argue that such an organization may help clinicians move beyond tic visibility alone when identifying the difficulties that matter most to the child and when defining individualized treatment targets. The framework is intended to support an initial inquiry across all three domains, with more detailed or quantitative assessment reserved for areas associated with clinically meaningful functional consequences.

## 2 Beyond tic severity: a three-domain clinical framework

The three domains may converge or diverge within the same child, while their clinical relevance is evaluated through transversal functional outcomes. The framework is summarized in Figure 1.

**Figure 1:**
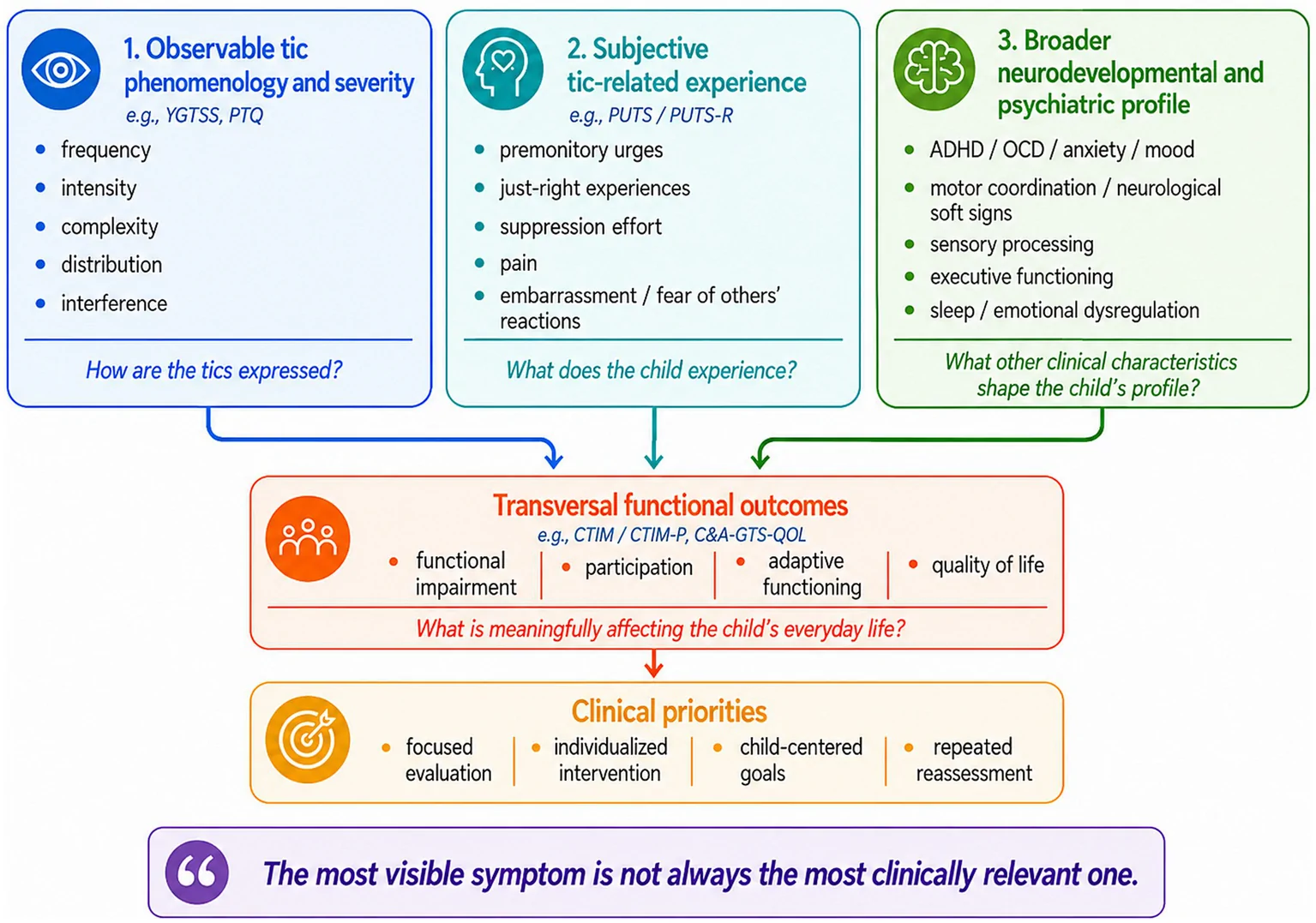
Three-domain clinical framework for multidimensional assessment in children with tic disorders. The framework distinguishes observable tic phenomenology and severity, subjective tic-related experience, and the broader neurodevelopmental and psychiatric profile. Functional impairment, participation, adaptive functioning, and quality of life are considered transversal outcomes that inform clinical prioritization and individualized treatment targets.

### 2.1 Observable tic phenomenology and severity

The first domain concerns the phenomenological characterization of motor and phonic tics, including their number, frequency, intensity, complexity, anatomical distribution, and interference. The Yale Global Tic Severity Scale (YGTSS) remains the most widely used clinician-rated instrument for this purpose and allows motor and vocal tics to be characterized separately while providing measures of overall tic severity and impairment (2, 9). Parent-reported measures such as the Parent Tic Questionnaire (PTQ) may provide complementary quantitative information on tic frequency and intensity (10). The YGTSS Total Tic Score primarily quantifies tic phenomenology and severity, whereas its impairment component captures the impact attributed to tics and should not be interpreted as a comprehensive measure of the child’s overall functioning, participation, adaptive functioning, or quality of life. This level of assessment is indispensable for confirming the nature of the phenomena, supporting differential diagnosis, establishing a baseline, and monitoring change over time.

However, tic expression is intrinsically variable. Frequency and intensity may fluctuate across the day and across settings according to fatigue, emotional arousal, attentional demands, social context, and opportunities for suppression (2). A clinical consultation therefore provides only a sample of the child’s tic repertoire and may substantially underestimate—or occasionally overestimate—its usual expression. Information from the child, parents, and school, together with longitudinal history and, when appropriate, brief video recordings obtained in everyday contexts, may provide a more representative picture (2).

Comparable tic severity may be associated with markedly different tic-related impact across children and contexts. Frequent and conspicuous tics may have limited functional consequences, whereas the functional impact of tics does not necessarily correspond to their visibility. This domain therefore addresses how tics are expressed, but not their overall functional consequences.

### 2.2 Subjective tic-related experience

The second domain shifts attention from the observable phenomenon to the child’s internal experience. Premonitory sensations may include tension, pressure, itching, a build-up of energy, feelings of incompleteness, or “not just right” experiences and are conceptually distinct from more persistent forms of sensory hypersensitivity (11, 12). Instruments such as the Premonitory Urge for Tics Scale and its revised version can support their assessment, although interpretation in younger children requires caution because body awareness, language, and metacognitive abilities influence the capacity to identify and describe internal sensations (11, 13). Limited reporting of premonitory sensations should therefore not automatically be interpreted as their absence.

Tic suppression is another relevant aspect of subjective experience. Suppressibility is not an all-or-none characteristic and varies according to the tic, context, attentional resources, and environmental contingencies (2, 7, 8). Experimental studies show that children and adolescents may markedly reduce observable tics during reinforced suppression without necessarily showing subsequent rebound or parallel reduction in premonitory sensations (8). For some children, suppressing tics may be experienced as effortful, draining, or interfering, and these effects may vary across individuals and contexts. The child’s perceived effort and any associated functional consequences should therefore be explored directly. Experimental studies, however, do not consistently demonstrate clinically meaningful attentional fatigue, impaired concentration, executive overload, or delayed functional consequences at the group level.

Pain and physical consequences should also be explored explicitly. Repetitive or forceful movements may lead to musculoskeletal discomfort, injury, headache, or local pain, while self-injurious tics or repeated vocalizations may produce additional physical distress. Pain is increasingly recognized as clinically relevant in Tourette syndrome but remains incompletely represented in commonly used tic outcome measures (14).

Subjective tic-related experience may also include embarrassment, stigma-related distress, fear of others’ reactions, and the perceived effort involved in managing tics. These experiences should be explored directly with the child, as they may not be readily inferred from observable tic expression.

### 2.3 Broader neurodevelopmental and psychiatric profile

The third domain concerns the broader neurological, neurodevelopmental, and psychiatric profile of the child. ADHD, obsessive-compulsive symptoms, anxiety, depression, emotional dysregulation, and sleep difficulties are well-established contributors to quality of life and daily functioning in tic disorders (2, 4, 5). Assessment should not be restricted to categorical diagnoses, as subthreshold difficulties may still affect learning, relationships, and self-regulation.

Initial assessment of this domain should be guided by the presenting concern. When psychiatric, behavioral, or executive difficulties are suspected, first-level assessment may include standardized screening questionnaires completed by parents and teachers, together with child or adolescent self-report when developmentally appropriate. Motor concerns may be explored through developmental history, a neurological soft-signs-driven examination, and caregiver-report screening of everyday motor difficulties when appropriate. Sensory concerns may be explored through focused clinical questioning and/or standardized caregiver-report screening of sensory processing. When first-level assessment identifies persistent or functionally relevant concerns, second-level evaluation should use standardized domain-specific instruments, such as neuropsychological tests, structured psychopathological assessment, standardized motor assessment, or formal sensory assessment, according to the area of concern.

Motor and neurological characteristics may contribute to the child’s difficulties independently of tic severity. Coordination, balance, motor planning, visuomotor integration, rhythm, or fine motor difficulties warrant further attention when they emerge from history or examination and are associated with functional consequences. Neurological soft signs—including overflow movements, dysrhythmia, subtle abnormalities of balance and gait, mild tone abnormalities, dysmetria, or other non-focal motor signs—are generally regarded as markers of neurodevelopmental variability rather than disorder-specific abnormalities (15). Motor and neurological soft-sign differences have been reported in children with tic disorders (16), but available evidence remains limited and does not establish a robust or disorder-specific phenotype. Their identification may nevertheless help characterize the broader neurodevelopmental profile and support targeted neurological or developmental assessment when clinically relevant.

Sensory processing deserves consideration when persistent hypersensitivity or difficulties in filtering or adapting to environmental stimuli extend beyond tic-specific premonitory urges (12). Further exploration is most relevant when these features are evident across contexts or interfere with daily activities or participation. Pediatric studies have associated sensory dysregulation with executive difficulties and comorbidity (17), while more recent data show heterogeneous relationships between sensory profiles, tic severity, and premonitory urges (18, 19). Current evidence does not establish consistent relationships between sensory-processing differences, tic severity, and premonitory urges, nor does it support a disorder-specific sensory phenotype; their independent contribution to functional outcomes also remains insufficiently defined.

Executive difficulties may also contribute to everyday impairment, particularly in the presence of ADHD. Meta-analytic evidence suggests that inhibitory-control differences in Tourette syndrome are generally small to moderate and strongly influenced by comorbid ADHD (20). More detailed assessment may be appropriate when difficulties involving attention, working memory, flexibility, planning, automatization, or self-regulation emerge from history, observation, or school functioning and affect daily performance or treatment planning. Whether adequate performance on structured tasks may in some children rely on increased attentional or compensatory resources remains insufficiently established. Routine neuropsychological testing is not indicated in all children with tics.

Importantly, abnormal neurological findings or reduced performance on standardized motor or neuropsychological measures do not in themselves establish clinically meaningful functional impairment. Their relevance should be judged in relation to the child’s everyday functioning and participation.

### 2.4 Functional consequences as transversal outcomes

The clinical relevance of findings across the three domains should ultimately be evaluated through their consequences in everyday life. In this framework, clinical burden is used as an overarching concept referring to meaningful real-life consequences associated with tic-related or other clinical features, rather than being inferred from symptom severity, examination findings, or performance deficits alone.

Several related constructs should therefore be distinguished. Symptom severity describes the characteristics of a symptom itself; neurological or neuropsychological performance refers to findings obtained through examination or standardized tasks; functional impairment concerns interference with age-appropriate activities and daily functioning; adaptive functioning refers to age-appropriate conceptual, social, and practical independence; participation concerns involvement in life situations; and health-related quality of life reflects perceived physical, emotional, social, and functional wellbeing. These constructs may overlap but are not interchangeable.

Functional impact should be assessed across informants and contexts, as children, parents, and school may provide complementary perspectives according to environmental demands, opportunities for suppression, and social responses. Child and parent reports should be considered jointly; discrepancies may be clinically informative, and self- and parent-proxy ratings of health-related quality of life are not fully interchangeable in adolescents with Tourette syndrome (21). Instruments such as the CTIM/CTIM-P can help distinguish tic-related from non-tic-related impairment across home, school, and social domains (22). Across all three domains, the central functional question is: “What is currently limiting this child’s everyday functioning, participation, wellbeing, or developmental opportunities?”

Functional consequences may arise from any of the three domains. Tics may interfere directly with school or social activities; premonitory sensations, pain, embarrassment, or suppression effort may contribute to distress, avoidance, or concealment; and psychiatric, motor, sensory, or executive characteristics may independently affect learning, relationships, autonomy, or participation. Functional impairment, participation, adaptive functioning, and quality of life should therefore be regarded as transversal outcomes rather than as components of the broader neurodevelopmental and psychiatric profile.

## 3 From multidimensional assessment to clinical priorities

The clinical value of the framework lies in translating assessment into prioritization. Assessment should remain selective rather than exhaustive: its purpose is not to generate a standardized battery for every child with tics, but to identify clinically relevant findings that may warrant more focused evaluation. When quantitative assessment is clinically indicated, measurement should be matched to the construct of interest. Tic severity may be assessed with the YGTSS, disease-specific quality of life with instruments such as the C&A-GTS-QOL (23), and adaptive functioning selectively when everyday independence is a specific concern. Attention, executive, emotional, or compulsive difficulties may prompt neuropsychological or psychopathological assessment; clumsiness or other motor abnormalities a broader neurological evaluation; sensory features a focused exploration; and difficulties in autonomy or participation a functional and contextual assessment.

Clinical priorities should be guided by three questions: which manifestations are present, which are associated with meaningful consequences in everyday functioning and participation, and which are currently modifiable? Mild, non-painful, minimally interfering tics may require only psychoeducation, monitoring, and environmental adjustments. When tics or tic-related experiences are associated with meaningful functional consequences, intervention may focus directly on these manifestations. When psychiatric, motor, sensory, executive, or contextual factors contribute more substantially to functional limitations, these may reasonably take precedence even while tics remain visible.

This approach requires shared and revisable decision-making. Children, parents, teachers, and clinicians may assign different importance to the same manifestations, and such discrepancies may provide clinically relevant information rather than simply represent disagreement. Treatment goals should therefore extend beyond tic reduction to include reduced pain, distress, avoidance, stigma, or effort and improved participation.

Because the clinical relevance of the three domains may change across development and contexts, reassessment should focus on whether the child is functioning better, not simply on whether tics are less frequent. The most appropriate treatment target is therefore not necessarily the most visible or severe manifestation, but the clinically modifiable factor most strongly associated with meaningful functional consequences for the child.

To facilitate clinical translation, the accompanying Supplementary Clinical Interview Guide (Supplementary material) is presented as a qualitative orientation tool for child- and caregiver-centered exploration of domains that may warrant further assessment. It is not a validated screening, diagnostic, severity, or scoring instrument and should not be interpreted as operationalizing or validating the framework.

## 4 Discussion: limitations and future directions

The proposed framework is intended as a clinical heuristic for organizing assessment and prioritization, not as a validated dimensional model, diagnostic algorithm, standardized score, or prescriptive pathway. Its purpose is to structure clinically relevant information while preserving the distinction between observable tic phenomenology, subjective tic-related experience, broader neurodevelopmental and psychiatric characteristics, and transversal functional outcomes.

The strength of evidence differs both with regard to whether these features are consistently observed in tic disorders and, more importantly, whether they make an independent contribution to functional impairment, participation, or quality of life. Psychiatric comorbidities are well-established contributors to these outcomes, whereas evidence concerning neurological soft signs, motor coordination, sensory processing, and possible compensatory executive costs is more limited or inconsistent.

The framework also requires empirical evaluation. Future studies should examine whether its use helps clinicians organize clinically relevant information across the three domains, identify priorities that may be overlooked by tic-severity measures alone, and support more consistent and individualized assessment and treatment planning. Qualitative studies could explore its acceptability, perceived usefulness, and applicability across clinical settings and developmental stages. Longitudinal research could then assess whether priorities identified through the framework are associated with functional outcomes, participation, quality of life, treatment response, and developmental trajectories. The framework may also inform outcome selection in treatment research by encouraging assessment beyond tic reduction alone and by helping investigators align outcome measures with observable tic severity, subjective tic-related experience, and the functional consequences most relevant to the intervention and study population.

The accompanying Supplementary Clinical Interview Guide (Supplementary material) should likewise be regarded as a qualitative orientation tool rather than a validated screening, diagnostic, severity, or scoring instrument. Its content was derived from the clinical domains discussed in this Perspective and from principles emphasized in contemporary tic-disorder assessment, including child- and caregiver-reported priorities, selective rather than indiscriminate assessment, contextual evaluation, and reassessment over time.

## 5 Conclusion

In children with tic disorders, the most visible symptom is not always the most clinically relevant one. Observable tic phenomenology, subjective tic-related experience, and broader neurodevelopmental and psychiatric characteristics may contribute differently to functional outcomes across development and contexts. Recognizing this complexity does not call for indiscriminate testing, but for selective assessment focused on the factors that meaningfully affect functioning, participation, wellbeing, and developmental opportunities. Treatment priorities should therefore be guided not by tic severity alone, but by the clinically modifiable factors most relevant to the child’s everyday life.

## Data Availability

The original contributions presented in the study are included in the article/Supplementary material, further inquiries can be directed to the corresponding author.
