# Supplementary material for: When tics are not the whole story: rethinking clinical assessment in children with tic disorders
Source: Front Neurol. 2026 Oct 21;17:1971225. doi: 10.3389/fneur.2026.1971225 (PMC13731256; doi:10.3389/fneur.2026.1971225)
Supplement: Supplementary file 1 [file Data_Sheet_1.docx]

**Clinical Interview Guide for Multidimensional Assessment in Children with Tic Disorders**

**Purpose and scope.** This qualitative clinical orientation tool supports child- and caregiver-centered exploration of clinically relevant domains. Its content reflects the three-domain clinical framework presented in the accompanying Perspective and principles of selective, contextual, and developmentally informed assessment.

| **CHILD** | **PARENT / CAREGIVER / SCHOOL** |
| --- | --- |
| “What is the thing that causes you the most difficulty or bothers you the most at the moment?” Response: ____________________________________ | “What difficulty currently has the greatest impact on the child’s everyday life?” Response: ____________________________________ |

**1. OBSERVABLE TIC PHENOMENOLOGY AND SEVERITY**

☐ Motor and/or vocal tics

☐ Frequency / intensity / complexity

☐ Distribution and contextual variability

☐ Observable interference

**Prompt:** *How are the tics expressed across different settings and situations?*

*Consider standardized tic-severity assessment when clinically indicated (e.g., YGTSS, PTQ).*

**2. SUBJECTIVE TIC-RELATED EXPERIENCE**

☐ Premonitory sensations / urges

☐ “Just-right” or incompleteness experiences

☐ Perceived effort associated with suppression

☐ Pain or physical discomfort

☐ Embarrassment, stigma, or fear of others’ reactions

**Prompt:** *What do you feel before a tic? Do you try to hold tics back? How difficult or effortful is this for you?*

*Consider targeted assessment when clinically indicated (e.g., PUTS/PUTS-R).*

**3. BROADER NEURODEVELOPMENTAL AND PSYCHIATRIC PROFILE**

| **Psychiatric / regulatory**  ☐ Attention / impulsivity / hyperactivity  ☐ Obsessive-compulsive symptoms  ☐ Anxiety / mood symptoms  ☐ Emotional / behavioral dysregulation  ☐ Sleep difficulties  *Examples: Conners rating scales; CBCL/ASEBA instruments; child/adolescent self-report measures when appropriate.* | **Neurological / motor**  ☐ Coordination, clumsiness, handwriting / fine motor difficulties  ☐ Overflow, dysrhythmia, neurological soft signs  ☐ Balance, gait, tone, other neurological concerns  *Examples: PANESS-oriented neurological examination; DCDQ or comparable caregiver-report motor screening tools.* | **Sensory / executive**  ☐ Sensory hypersensitivity or sensory-seeking  ☐ Sensory experiences distinct from tic-specific urges  ☐ Attention, working memory, flexibility, planning, self-regulation  *Examples: Sensory Profile 2 or comparable sensory-processing questionnaires; BRIEF-2 or comparable parent/teacher executive-function rating scales.* |
| --- | --- | --- |

**Prompt:** *Do first-level findings suggest psychiatric, motor, sensory, or executive difficulties that may require further assessment?*

*If so, consider standardized second-level psychopathological, neuropsychological, motor, or sensory assessment according to the presenting concern.*

**4. TRANSVERSAL FUNCTIONAL OUTCOMES**

☐ School functioning / learning

☐ Family life and peer relationships

☐ Leisure, sport, and social participation

☐ Adaptive functioning / everyday independence

☐ Avoidance, concealment, stigma, or reduced opportunities

☐ Health-related quality of life

**Prompt:** *What is the child currently unable to do, avoiding, or doing with disproportionate difficulty or effort?*

*When quantitative assessment is required, select measures according to the construct of interest (e.g., CTIM/CTIM-P for tic-related versus non-tic-related impairment) rather than inferring functional impact from symptom-severity or performance measures alone.*

**ORIENTATIVE CLINICAL SUMMARY**

1. Manifestations currently present: ______________________________________________

2. Manifestations associated with meaningful consequences in everyday functioning and participation: ______________________________________________

3. Currently modifiable factors / potential treatment targets: ______________________________________________

Child / family priorities: ______________________________________________

Information to integrate across settings: ______________________________________________

**Abbreviations.** YGTSS = Yale Global Tic Severity Scale; PTQ = Parent Tic Questionnaire; PUTS = Premonitory Urge for Tics Scale; PUTS-R = Premonitory Urges for Tic Disorders Scale-Revised; CBCL = Child Behavior Checklist; ASEBA = Achenbach System of Empirically Based Assessment; PANESS = Physical and Neurological Examination for Subtle Signs; DCDQ = Developmental Coordination Disorder Questionnaire; BRIEF-2 = Behavior Rating Inventory of Executive Function, Second Edition; CTIM = Child Tourette Syndrome Impairment Scale; CTIM-P = Child Tourette Syndrome Impairment Scale-Parent Report.

**Note.** This guide is a qualitative clinical orientation tool. It is not a validated screening, diagnostic, severity, scoring, or decision-making instrument and should not be interpreted as operationalizing or validating the proposed framework. It does not imply that all domains require formal testing in every child. Assessment should remain selective, developmentally informed, contextual, and guided by the child’s current difficulties and priorities.
